# Endotracheal intubation of COVID-19 patients by paramedics using a box barrier: A randomized crossover manikin study

**DOI:** 10.1371/journal.pone.0248383

**Published:** 2021-03-31

**Authors:** Oren Feldman, Nir Samuel, Noa Kvatinsky, Ravit Idelman, Raz Diamand, Itai Shavit

**Affiliations:** 1 Pediatric Emergency Department, Rambam Health Care Campus, Haifa, Israel; 2 Department of Neurobiology, Weizmann Institute of Science, Rehovot, Israel; Imam Abdulrahman Bin Faisal University College of Medicine, SAUDI ARABIA

## Abstract

**Background:**

In the prehospital setting, endotracheal intubation (ETI) may be required to secure the coronavirus disease 2019 (COVID-19) patient airway. It has been suggested that the use of a protective barrier can reduce possible aerosol delivery from patients to clinicians during ETI. We sought to assess the performance of ETI by paramedics wearing personal protective equipment with and without the use of a box barrier.

**Methods:**

A randomized, crossover simulation study was performed in a simulation laboratory. Study participants were 18 paramedics actively working in the clinical environment. Participants’ performance of ETI via direct laryngoscopy (DL) with and without the use of a box barrier was assessed. The sequence of intubation was randomized to either BoxDL-first or DL-first. The primary outcome was the success rate of ETI on first-attempt. The secondary and tertiary outcomes were ETI success rates on three attempts and total intubation time, respectively.

**Results:**

There were no differences between the DL group and the BoxDL group in one-attempt success rates (14/18 vs 12/18; P = 0.754), and in overall success rates (16/18 vs 14/18; P = 0.682). The mean (standard deviation) of the total intubation times for the DL group and the BoxDL group were 27.3 (19.7) seconds and 36.8 (26.2) seconds, respectively (P < 0.015).

**Conclusions:**

The findings of this pilot study suggest that paramedics wearing personal protective equipment can successfully perform ETI using a barrier box, but the intubation time may be prolonged. The applicability of these findings to the care of COVID-19 patients remain to be investigated.

## Introduction

The response of the Emergency Medical System (EMS) is an important factor in the fight against the coronavirus disease 19 (COVID-19) pandemic [1, 2]. The majority of critically ill patients with COVID-19 require respiratory support [3]. In the prehospital setting, endotracheal intubation may be required to secure a patient’s airways is some of the cases. In Paris, out of 300 consecutive COVID-19 patients, 18 (6%) were treated by endotracheal intubation by the Paris Fire Brigade Advanced Life Support teams [4].

Endotracheal intubation, however, is an aerosol-generating procedure and imposes a potential risk for aerosol-based transmission [5–7]. To reduce possible aerosol delivery from patients to clinicians during airway management, several types of protective barriers, such as plastic boxes, tents, hoods or canopies, have been suggested as a cost-effective solution. These protective barriers to be used in the pre-hospital and in-hospital settings were not designed to replace personal protective equipment (PPE) but to provide another layer of protection [8–14].

In this simulation study, we aimed to assess the performance of endotracheal intubation by paramedics wearing PPE with and without the use of a box barrier.

## Materials and methods

### Study design

A randomized, crossover simulation study was performed at the simulation laboratory of a tertiary hospital medical center. We compared participants’ performance of endotracheal intubation via Direct Laryngoscopy (DL) with and without the use of a box barrier. The procedure was performed according to recommendations for patients with COVID-19 infection, including PPE use [15, 16]. The Institutional Review Board of Rambam Health Care Campus waived the need for ethics approval as this was considered a quality control initiative.

### Study participants

Study participants were paramedics employed by the national EMS organization, actively working in the clinical environment, who volunteered to participate in the study. All the paramedics had prior experience with tracheal intubation in the prehospital setting, using DL. None of them performed endotracheal intubation of a COVID-19 patient, and none was familiar with the use of a box barrier. The sequence of intubation was randomized to either BoxDL-first or DL-first. Using a computerized random-number generator, an allocation sequence was created and course participants were divided into two groups for the study: BoxDL-first and DL-first.

### Study instrument

**Fig 1:**
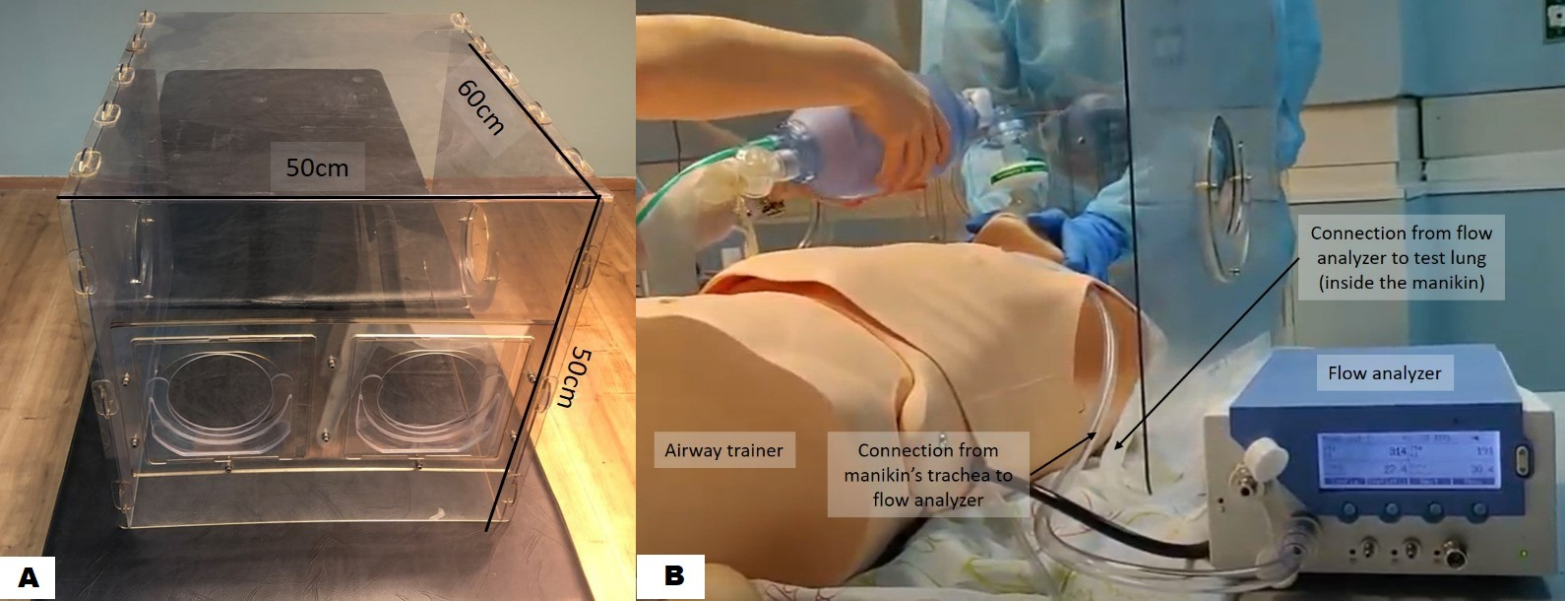
Study model. A. Study instrument, polycarbonate barrier box. B. Ventilation of the manikin using a two-person technique. The participant is holding the facemask while concurrently a study investigator is activating the Ambu®-Bag.

The box barrier (Palram Ltd, Ramat Yohanan, Israel) is a polycarbonate box, with a low manufacturing cost, designed for self-assembly (Fig 1A). The dimensions of the rear panel are 50 X 50 cm, and the box length is 60 cm. The patient’s head can be reached via two 12 X 12 cm round holes in the rear panel. Each of these holes is located within a square with lateral movement of a few centimetres to allow more freedom for the provider’s hand movements without enlarging the hand holes. Additional 12 X 12 cm holes are located on each side of the lateral panels, to allow airway assistant help (Fig 1A).

### Study model

A Laerdal Deluxe Airway Trainer (Laerdal Medical AS, Stavanger, Norway) was used for ETI. The box was placed over the manikin’s head which was placed in a neutral position. Ventilation was performed using a two-person technique according to recommendations for patients with COVID-19 infection [15], and in accordance with national and local institutional guidelines. In two-person technique, when bag-valve-mask ventilation is performed, one paramedic maintaining a two-handed grip on the mask to ensure a tight seal while the other paramedic squeezes the bag [15]. The participant held the facemask of the Ambu®-Bag on the manikin while concurrently a study investigator was activating the Ambu®-Bag (Fig 1B). To optimally mimic ventilation in-vivo, we assembled a system that measured tidal volumes during ventilation [17]. A test lung (EasyLung™, compliance 25 ml/mbar, resistance 20 mbar/L/s, maximal volume 1000 ml IMT Medica, Buchs, Switzerland) was placed inside the manikin’s chest, replacing the original manikin’s lungs and not visible from the outside. The test lung was connected to a flow analyzer (PF-300, IMT Analytics AG, Buchs, Switzerland) which was connected through a sealed tube to the manikin’s trachea. The flow analyzer was located on a nearby table and was visible to a second study investigator (Fig 1B) [17]. For the intubation, the participant inserted his/her hands through the rear holes of the barrier box, and received the laryngoscope and the tracheal tube from the study investigator through a side hole.

### Study procedure

Prior to the study, participants received a 45-min lecture on the treatment of COVID-19 patients with respiratory failure, followed by an educational video on the use and the technique of endotracheal intubation using the box barrier [15, 16, 18]. During this training, each participant performed one successful endotracheal intubation via DL on an airway model (Laerdal Airway Trainer, Laerdal Medical AS, Stavanger, Norway) which was different from the one used in the study. Thereafter, participants were randomly divided into the two study groups.

**Fig 2:**
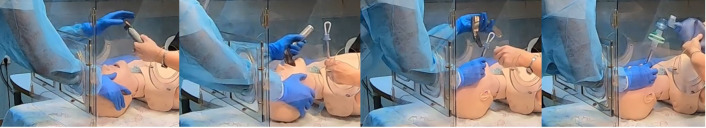
Endotracheal intubation using the barrier box.

Before entering the study room in which the study model was placed, each participant donned the following PPE: N95 respirator, face shield, gown, and gloves. In the study room each participant was asked to perform intubation via DL with and without the box in the sequence determined by randomization. Each procedure was recorded by two cameras. Recording began with the first endotracheal intubation attempt and ended when successful intubation was confirmed. Data for analysis were obtained from the recorded videos. Endotracheal intubation was comprised of four steps which were later timed in the recorded videos (Fig 2).

The participant began by asking for a laryngoscope which was handed to him by an assistant (one of the investigators, a nurse). A direct laryngoscope with size 3 Macintosh blade was provided through the box’s side hole. When ready for tube insertion, a 7.0 mm cuffed tube with stylet was handed to him/her through the box’s side hole. At that moment, the investigator started counting loudly the time elapsing, to verify that no more than the 30 seconds allowed between the beginning of tube insertion until end of intubation attempt, as per the recommendations for endotracheal intubation of COVID-19 patients [16]. At the end of tube insertion, the participant asked the assistant to inflate the balloon, connect the Ambu®-Bag and start ventilations (Fig 2).

The procedure was ended by the participant when he saw a chest rise. A second study investigator confirmed tube placement by verifying presence of tidal volumes in the flow analyzer. If the tube was not in place, the participant was asked by the investigator to perform another intubation attempt. If the participant did not complete the intubation attempt in 30 seconds, he was asked to put the face-mask on so that Ambu-bagging by the study investigator could start over again (Fig 1B). A maximum of three intubation attempts were allowed.

Following a 2-minute rest period, the participant was asked to perform the second intubation. Intubation without a box followed a similar sequence.

### Study outcome measures

#### Primary outcome measure–first-attempt success rate

The primary outcome for the study was the success rate of endotracheal intubation on the first attempt. Success was defined by lung inflation as indicated by the flow analyzer [16].

#### Secondary outcome measure–overall intubation success rate

The secondary outcome measure was intubation success rate on three attempts [19].

#### Tertiary outcome measure–total intubation time

The total intubation time was the sum of intubation attempt times [20–22]. Intubation attempt time was defined as the time from the start of the intubation attempt (participant has the laryngoscope in his hands) until intubation success is confirmed, or 30 seconds have elapsed.

#### Ease of endotracheal intubation using the box barrier

Following the two study procedures, participants were asked to record their impressions of ease-of-intubation using the box barrier on a five-point Likert Scale (“Endotracheal intubation using the box barrier was easy”; 1—strongly disagree, 2—disagree, 3—neither agree nor disagree, 4—agree, 5—strongly agree). Data were collected anonymously.

### Statistical analysis

Descriptive statistics were generated, including medians and interquartile ranges (IQR), means and standard deviations (SD). As this was a crossover trial, pairing was taken into account in the statistical analysis. McNemar’s test was used for comparing the success rate of intubation with and without the box, and Wilcoxon signed ranks test was used to compare intubation times between the two groups. All statistics were calculated using the StatsDirect statistical software (v2.6.6, StatsDirect Limited, Cheshire, UK).

## Results

Eighteen paramedics with a median age of 27.5 years (IQR 25–30) participated in the study. The median professional experience was 5 years (IQR 4–8). There were no differences between the DL group and the BoxDL group in one-attempt success rates (14/18 vs 12/18; P = 0.754), and in overall success rates (16/18 vs 14/18; P = 0.682). The mean (SD) of the total intubation times for the DL group and the BoxDL group were 27.3 (19.7) seconds and 36.8 (26.2) seconds, respectively (P < 0.015) (Table 1). The overall ease of intubation using the barrier box was 3 (IQR 2–4). The ease of intubation of the DL-first group was 3 (IQR 3–4). The ease of intubation of the BoxDL-first group was 2 (IQR 2–4).

**Table 1:** Comparison of intubation success rates and duration between the two study groups.

	Endotracheal intubation with DL	Endotracheal intubation with DL using a protective box	P value
**Success rates on first attempt (%)**	14/18 (77.8)	12/18 (66.7)	NS
DL first	7/9 (77.8)	6/9 (66.7)	NS
Box-DL first	7/9 (77.8)	6/9 (66.7)	NS
**Overall Success rates (%)**	16/18 (88.9)	14/18(77.8)	NS
DL first	8/9 (88.9)	8/9 (88.9)	-
Box-DL first	8/9 (88.9)	6/9 (66.7)	NS
**Total intubation time, mean ± SD (sec)**	27.3 ± 19.7	36.8 ± 26.2	0.015
DL first	27.9 ± 16.3	44.6 ± 32.8	NS
Box-DL first	26.9 ± 23.7	26.3 ± 7.3	NS

Notes

DL = Direct Laryngoscopy, SD = Standard Deviation, NS = Not Significant

## Discussion

Since EMS personnel have a major role in fighting the COVID-19 pandemic, it is crucial that they safely treat critically ill patients [1, 2]. Endotracheal intubation seems to be the highest risk procedure [5–7]. Recent studies have demonstrated the potential benefit of using a barrier device to protect against infection during tracheal intubation [6–8, 11].

The main finding of the current simulation study is that, in using DL, paramedics wearing PPE had similar intubation success rates with and without the use of a barrier box. The overall intubation success rates and the success rates on first-attempt were similar for both groups, and were in the range of 70–90 percent. These findings suggest that with proper training, paramedics who are inexperienced with box barrier use, are able to successfully perform tracheal intubation using the box while wearing PPE.

These findings are corroborated by similar studies that examined the success rates of anesthesiologists using videolayngoscopy [18, 20]. In one study, eight anesthesiologists wearing PPE performed endotracheal intubation on an airway model using four videolaryngoscopy devices, and a direct laryngoscope. Success rates were 100 percent with each of the laryngoscopes except with the Airtraq® AVANT videolaryngoscope (85.7 percent) [18]. In another study with 12 anesthesiologists as participants, first-attempt success rates using videolaryngoscopy was 10/10 without the barrier box, and 9/12 and 10/12 with a barrier box [20]. These results are in the range of values reported in our study.

We found that intubation time with DL was almost 1.4 times longer with the barrier box compared to no box (37 sec vs 27 sec). Prolonged intubation times with the use of a barrier box were also reported when anesthesiologists and intensivists performed the procedure with videolaryngoscopy. In these studies, the duration of intubation time was 1.1–1.8 longer with the protective box compared to no box [20, 21].

The prolongation of intubation time seems to be an important finding. The increased duration of intubation shown, although not relevant in a manikin study, may interfere with endotracheal intubation in a real situation. A prospective study with real patients is required to evaluate its translation to airway management on COVID-19 patients.

On a Likert scale of 1 to 5, participants rated the ease of intubation with the use of a box as 3; neither agree nor disagree. Participants of the DL-first group disagreed with the statement that intubation was easy. These findings suggest that participants felt less comfortable with the barrier box despite the training they received prior to the study, a finding that is consistent with the results of a recent study [21].

Our study has several limitations. Firstly, given the difficulty in recruiting active paramedics to the study during times of the pandemic, the sample size in this pilot study was relatively small, and a power analysis was not conducted prior to conducting the study. Secondly, we did not examine other types of protective barriers such as tents, hoods, or canopies. Thirdly, this was a simulation study; therefore, the applicability of the data to real-life scenarios is unknown. Fourthly, most data regarding the ability of barrier-enclosure systems such as the box barrier used in our study to contain or limit aerosols, is of low-level evidence [23].

In conclusion, the findings of this pilot study suggest that paramedics wearing PPE can successfully perform endotracheal intubation using the barrier box but the intubation time may be prolonged. The applicability of these findings to the care of COVID-19 patients remain to be investigated.

## Abbreviations

EMS: Emergency Medical System
PPE: Personal Protective Equipment
DL: Direct Laryngoscopy
COVID-19: Coronavirus Disease 19
